# Supplementary material for: First-in-human trial of blood–brain barrier opening in amyotrophic lateral sclerosis using MR-guided focused ultrasound
Source: Nat Commun. 2019 Sep 26;10:4373. doi: 10.1038/s41467-019-12426-9 (PMC6763482; doi:10.1038/s41467-019-12426-9)
Supplement: Supplementary file 1 — Supplementary Information [file 41467_2019_12426_MOESM1_ESM.pdf]

Supplementary Information

First-in-human Trial of Blood-Brain Barrier Opening in Amyotrophic Lateral Sclerosis using MR-Guided Focused Ultrasound

Abrahao and Meng et al.

SUPPLEMENTARY FIGURES

Supplementary Figure 1 Target placement for each subject overlapped onto intraprocedural axial T2-weighted MRIs.

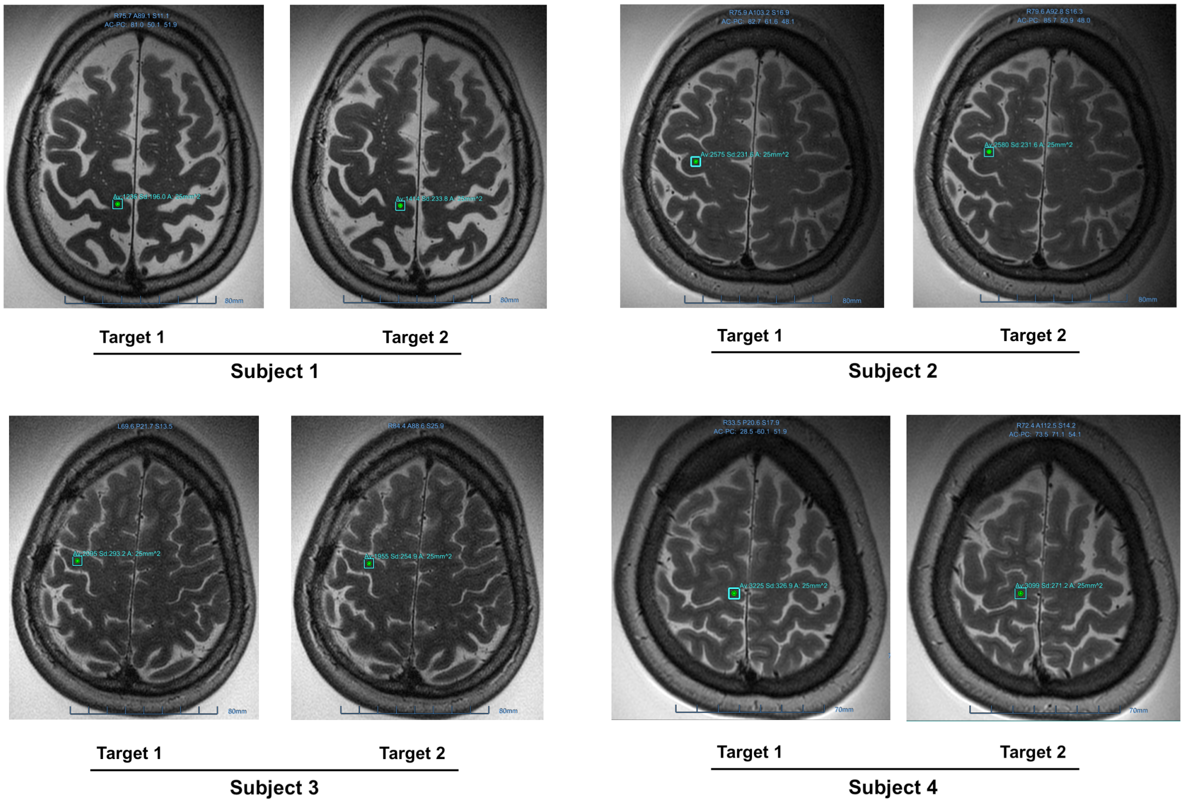

Each green box represents the target with 5 mm sides, centred over the arm or leg area as mapped by task fMRI.

Supplementary Figure 2 Serial comparisons of (A) FLAIR, (B) T2, (C) T2* gradient echo (GRE), and (D) DWI sequence MR images for subject four.

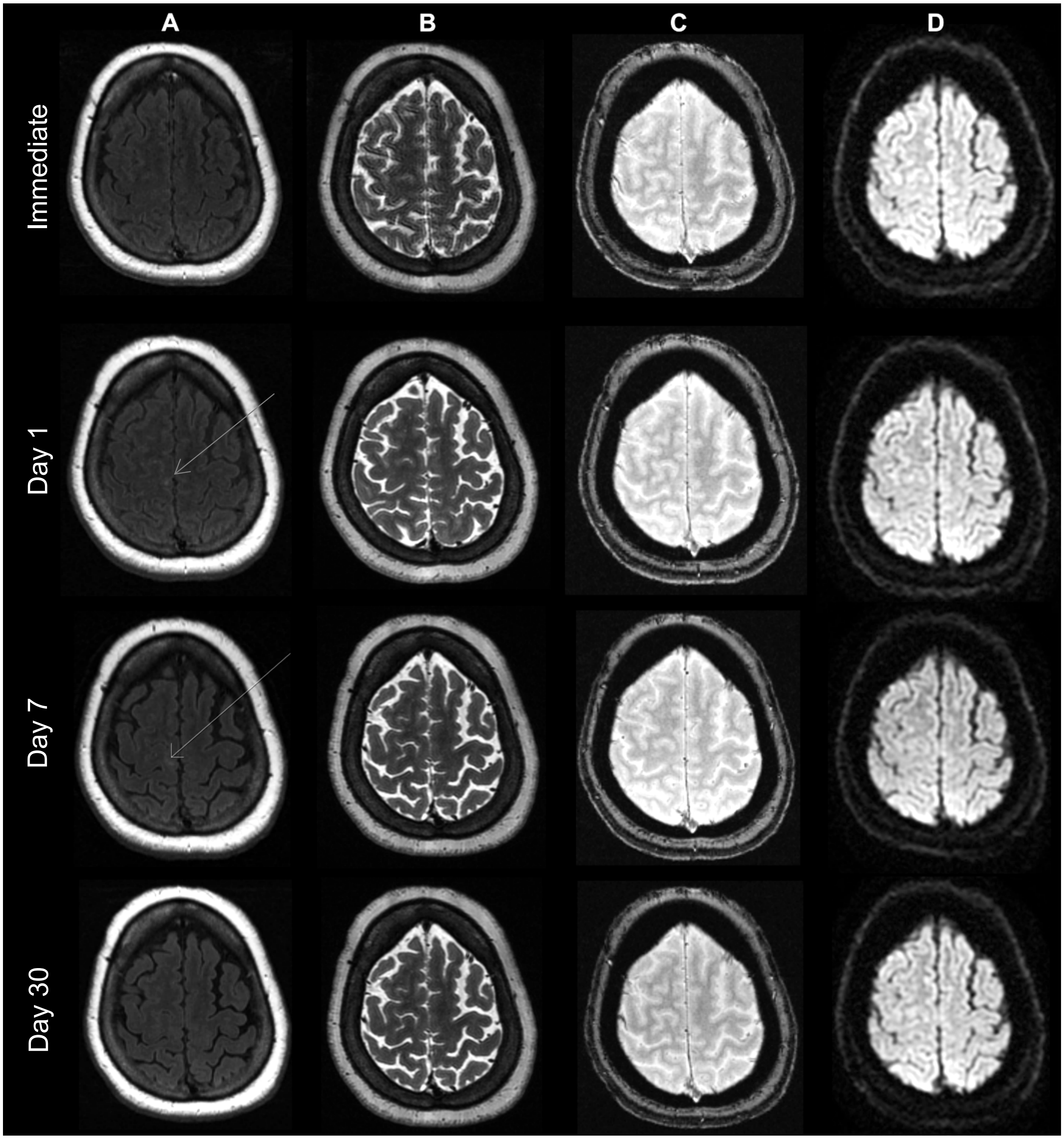

The arrow points to a hyperintense FLAIR signal in the sonicated region. This change was detected on day 1 and 7 after the procedure and resolved on day 30. No changes on T2, T2* gradient echo (GRE), or diffusion-weighted imaging (DWI) were detected.

Supplementary Figure 3 Longitudinal analysis of structural T1-weighted MRIs obtained throughout the study showed no significant cortical volume change of the left (filled circles) or right (open circles) precentral gyrus.

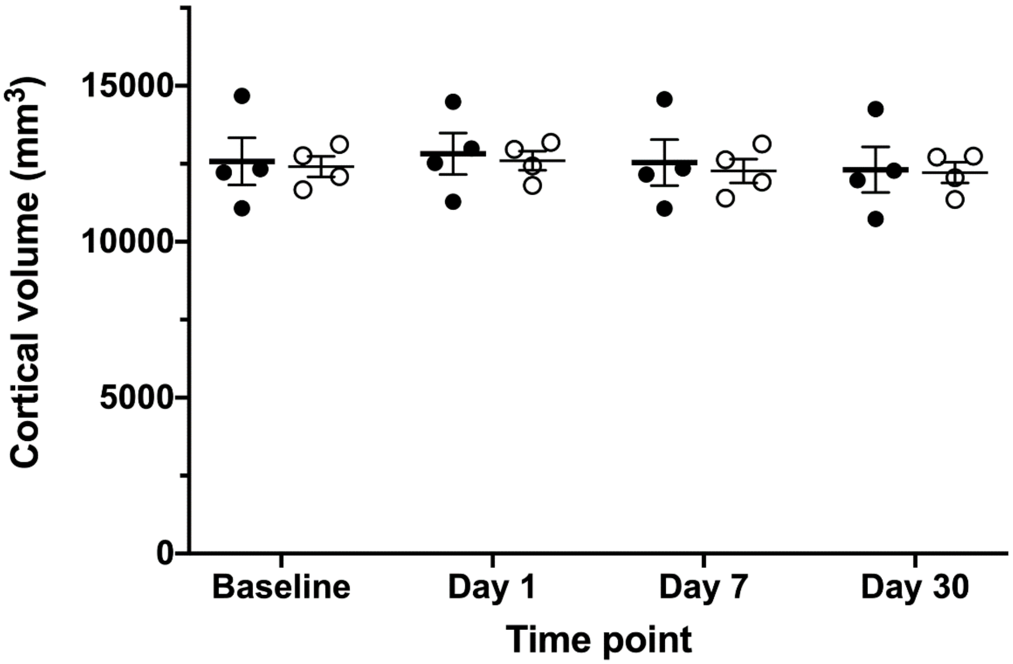

Error bars represent standard error of the mean. Raw data are provided in the Source Data file.

Supplementary Figure 4 Schematic diagrams of MRgFUS target and sonications

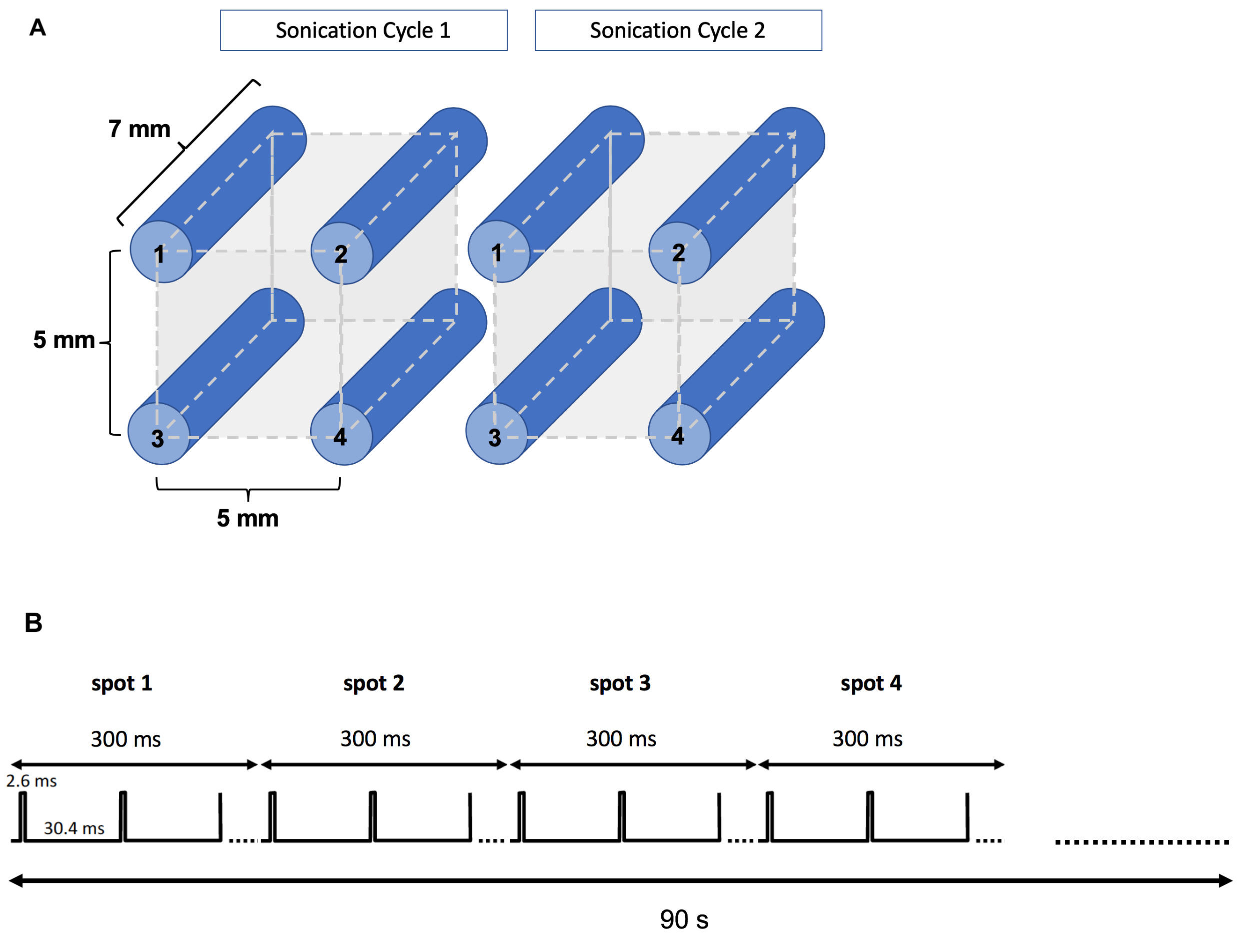

(A) Two 175 mm^3 volumes or targets were placed over the primary arm or leg region mapped by task fMRI. Each target consists of four spots (marked) the device sonicates sequentially, switching every 300 ms, as described in (B). Ultrasound was delivered with pulse repetition frequency of 0.3 Hz and duty cycle of 7.88%. Each sonication cycle (total duration of 90 seconds) was performed with an intravenous injection of 4 μl/kg perflutren microbubbles (Definity®, Lantheus Medical Imaging, USA).

SUPPLEMENTARY TABLES

Supplementary Table 1 Ultrasound parameters used for MRgFUS induced opening of the BBB in four subjects

	Total volume of target (mm^3)	Sonication power range (Watts)	Total number of 90-second sonications
**Subject 1**	350	6–10	3
**Subject 2**	350	4–8	2
**Subject 3**	350	8–10	4
**Subject 4**	350	4–7	3

For reference, 5 W of sonication power delivered by the clinical system at 220 kHz through an ex vivo human skull translated to 500 kPa measured by hydrophone at the center of the skull[1].

Supplementary Table 2 Intraprocedural medications administered and indications

	Intravenous Medication	Total Dose	Indication
**Subject 1**			
	Midazolam	1.5 mg	Anti-anxiety
	Fentanyl	100 mcg	Headache/pin pain prophylaxis
**Subject 2**			
	Odansetron	4 mg	Nausea prophylaxis
	Fentanyl	60 mcg	Pain prophylaxis
	Ketamine	15 mg	Sedation
**Subject 3**			
	Lorazepam	0.1 mg	Anti-anxiety
	Fentanyl	20 mcg	Headache/Pain
**Subject 4**			
	Midazolam	0.5 mg	Anti-anxiety
	Hydromorphone	0.3 mg	Pain at the pin site

Supplementary Table 3 Longitudinal clinical measures at baseline and post MRgFUS BBB opening

		Post-MRgFUS motor cortex BBB opening				
	Baseline	Immediate	Day 1	Day 7	Day 30	Day 60§
**Subject 1**						
MRC sum score*						
Left leg**	11	10	11	12	13	
Other limbs	60	59	60	60	60	
ALSFRS-R total score	32				30	30
MoCA	26				27	
Modified Ashworth	0				0	
**Subject 2**						
MRC sum score						
Left arm**	8	12	8	10	11	
Other limbs	46	55	46	46	46	
ALSFRS-R total score	29				29	29
MoCA	30				30	
Modified Ashworth	0				0	
**Subject 3**						
MRC sum score						
Left arm**	12	12	13	12	12	
Other limbs	75	75	75	75	75	
ALSFRS-R total score	32				32	32
MoCA	27				28	
Modified Ashworth	0				0	
**Subject 4**						
MRC sum score						
Left Leg**	6	6	6	6	6	
Other limbs	66	66	66	64	66	
ALSFRS-R total score	30				30	30
MoCA	25				25	
Modified Ashworth	0				0	

* Medical Research Council (MRC) manual muscle strength rating for left-sided shoulder extensors, elbow flexors, elbow extensors, finger extensors, thumb flexor at the interphalangeal joint, abductor of the index finger and thumb abductors, hip flexors, hip abductors, knee extensors, knee flexors, ankle dorsiflexors and plantar flexors. Normal MRC sum score is 35 for each arm and 30 for each leg. ** indicates the limb contralateral to the sonicated motor cortex. § remote visit over the phone.

Supplementary Table 4 Potential risks associated with the transcranial application of MRgFUS

Category	Potential risks
**Scalp in the sonication pathway**	Skin burns (>2°) with ulceration of the skin<br>Scar formation<br>Loss of sensation<br>Atrophy
**Bone in the sonication pathway**	Bone necrosis
**Dura, venous sinus, and cortical veins**	Subdural bleeding<br>Vein thrombosis<br>Cortex heating<br>Seizures<br>Symptoms from disturbances of eloquent cortical areas (e.g. motor, sensory, auditory, visual, speech)
**Cerebral arteries**	Bleeding<br>Coagulation thrombosis<br>Vasospasm<br>Death
**Other brain tissue**	Necrosis of normal tissue due to incorrect targeting<br>Thermal damage to adjacent functional brain tissue (e.g. optical tract)<br>Cerebral infarction<br>Moderate or severe increase in cerebral edema as shown by MRIs<br>Symptomatic increase of intracranial pressure<br>Death

SUPPLEMENTARY METHODS

Inclusion Criteria

1. Diagnosed with laboratory-supported probable, clinically probable or definite ALS according to the World Federation of Neurology Revised El Escorial criteria
2. Right-hand dominant male or female aged 18 years or older
3. Capable of providing informed consent and complying with study procedures, including tolerability in the supine position and MRI examination without significant claustrophobia.
4. If taking riluzole, on a stable dose for at least 30 days prior to Screening Visit.
5. Slow Vital Capacity equal to or more than 50% predicted value for gender, height and age in the 30 days prior to the Screening Visit and able to lie supine without BiPAP.
6. Severe left arm weakness and functional impairment, defined as Medical Research Council muscle strength score equals 3 or less in the index finger abduction and thumb abduction on the left side; *OR* severe left leg weakness and functional impairment, defined as Medical Research Council muscle strength score equals 3 or less at the hip flexors and ankle dorsiflexors on the left side.
7. Able to communicate during the ExAblate® MRI-guided FUS procedure.

Exclusion Criteria

1. Unable to complete high-density CT and MRI studies of the head at the Screening Visit or any other MRI contraindication, such as:
   - Large body habitus and not fitting comfortably into the scanner
   - Difficulty lying supine and still for up to 3 hours in the MRI unit or significant claustrophobia
2. MRI findings:
   - Active infection/inflammation
   - Acute or chronic brain haemorrhages, specifically lobar or subcortical microbleeds, siderosis or macrohaemorrhages
   - Tumour/space occupying lesion
   - Meningeal enhancement
3. More than 30% of the skull area traversed by the sonication pathway is covered by scars, scalp disorders (e.g., eczema), or atrophy of the scalp
4. Clips or other metallic implanted objects in the skull or the brain, except shunts
5. Significant cardiac disease or unstable hemodynamic status including:

- Documented myocardial infarction within six months of enrollment
- Unstable angina on medication
- Unstable or worsening congestive heart failure
- Left ventricular ejection fraction below the lower limit of normal
- History of a hemodynamically unstable cardiac arrhythmia

- Cardiac or phrenic pacemaker
- Known right-to-left, bidirectional, or transient right-to-left cardiac shunt
- Patients with relative contraindications to perflutren including subjects with a family or personal history of QT prolongation or taking concomitant medications known to cause QTc prolongation,
- QT prolongation observed on screening ECG (QTc > 450 for men and >470 for women)

6. Uncontrolled hypertension (systolic > 150 or diastolic BP > 100 on medication)
7. On medications that increase the bleeding risk, specifically: a) aspirin or another antiplatelet medication (clopidogrel, prasugrel, ticlopidine, abciximab) for the last 7 days prior to treatment; b) oral, subcutaneous or intravenous anticoagulant medications, such as oral vitamin K inhibitors for the last 7 days, non-vitamin K inhibitor oral anticoagulant (dabigatran, apixaban, rivaroxaban) for the last 72 hours, and intravenous or subcutaneous heparin-derived compounds for the last 48 hours
8. History of a bleeding disorder, coagulopathy or a history of spontaneous haemorrhage
9. Known frontotemporal dementia
10. Abnormal coagulation profile, specifically: platelet <100,000/μl, Prothrombin Time >14 seconds, activated partial thromboplastin time (aPTT) >36 seconds, and INR > 1.3
11. Known cerebral or systemic vasculopathy, specifically cerebral amyloid angiopathy or systemic or central nervous system vasculitis.
12. Known auto-immune condition with or without neurological manifestations (e.g., multiple sclerosis (MS), systemic lupus erythematosus (SLE), Rheumatoid arthritis).
13. Current or planned use of oral, intramuscular or intravenous steroid drugs (such as prednisone, prednisolone, dexamethasone, triamcinolone, methylprednisolone, oxandrolone, and others) or immunosuppressant drugs (azathioprine, mycophenolate, tacrolimus, sirolimus, cyclophosphamide, and others) for more than 7 days
14. Known sensitivity/allergy to gadolinium (an alternative product may be used), DEFINITY® contrast or any of its components
15. Untreated, uncontrolled sleep apnea
16. Impaired renal function with cystatin C-based estimated glomerular filtration rate <30 mL per min per1.73m^2 and acute renal injury.
17. Currently in a clinical trial involving an investigational product or non-approved use of a drug or device.
18. Known respiratory diseases, specifically: chronic pulmonary disorders e.g., severe/uncontrolled COPD, pulmonary vasculitis, or other causes of reduced pulmonary vascular cross-sectional area, asthma or hay fever.
19. Patients with a history of drug allergies or multiple allergies where the benefit/risk of administering DEFINITY® is considered unfavourable by the study physicians in relation to the product monograph for DEFINITY®.
20. Unqualified fit for the anaesthesia by an anesthesiologist assessment, ASA I-III.

SUPPLEMENTARY NOTES

Supplementary Note 1 Anticipated Treatment Adverse Events from MRgFUS

All adverse events will be reported and analyzed for their relation to the ExAblate device as well as other causes (e.g. Expected procedure findings, Drug/Contrast media reactions, Medical conditions, and Unrelated to device or procedure). Adverse events related to the device or procedure are expected to be acute, and occur during or shortly after the procedure. Based on existing treatment experience in the brain using the ExAblate Neuro Type 1.0 (650 KHz), the following anticipated side effects have been identified as possibly occurring during/following the ExAblate procedure. In addition to severity as described by the Common Terminology Criteria for Adverse Events (CTCAE), adverse events are classified as procedure-related, device-related, blood-brain barrier disruption related, and unrelated.

Anticipated procedure-related events include:

- Claustrophobia
- Pain/discomfort related to fasting, placement of the stereotactic frame, immobility or position on the MRI table, and sonications
- Hypertension/hypotension, and bradycardia/tachycardia may be related to pre-procedure nervousness or comorbid conditions / missed medication dose pre-procedure as an NPO requirement
- Misalignment or reflection of the ExAblate beam resulting in mis-targeting
- Heating of structures in the ultrasound backbeam or forebeam
- Neurological deficits may result from damage to nearby structures in the brain and thus is specifically related to the target location. To date, the following has been reported from neuroablation by ExAblate Neuro Type 1.0 device: sensory or motor disturbances, ataxia or dysmetria, dizziness, dysphagia, and visual disturbances. These adverse events can be either transient or permanent
- Events unrelated to the ExAblate device, such as those related to an intravenous line or comorbid conditions

Additionally, the following side effects are thought to be improbable, but their relative risks remain to be defined by this and future studies.

SUPPLEMENTARY REFERENCE

1. Huang, Y., Alkins, R., Schwartz, M. L. & Hynynen, K. Opening the Blood-Brain Barrier with MR Imaging-guided Focused Ultrasound: Preclinical Testing on a Trans-Human Skull Porcine Model. *Radiology* **282**, 123–130 (2017).
