# Supplementary material for: First-in-human trial of blood–brain barrier opening in amyotrophic lateral sclerosis using MR-guided focused ultrasound
Source: Nat Commun. 2019 Sep 26;10:4373. doi: 10.1038/s41467-019-12426-9 (PMC6763482; doi:10.1038/s41467-019-12426-9)
Supplement: Supplementary file 3 — Reporting Summary [file 41467_2019_12426_MOESM3_ESM.pdf]

nature research

Corresponding author(s): Agessandro Abrahao

Last updated by author(s): Aug 7, 2019

Reporting Summary

Nature Research wishes to improve the reproducibility of the work that we publish. This form provides structure for consistency and transparency in reporting. For further information on Nature Research policies, see Authors & Referees and the Editorial Policy Checklist.

Statistics

For all statistical analyses, confirm that the following items are present in the figure legend, table legend, main text, or Methods section.

n/a	Confirmed	
☐	☒	The exact sample size (*n*) for each experimental group/condition, given as a discrete number and unit of measurement
☐	☒	A statement on whether measurements were taken from distinct samples or whether the same sample was measured repeatedly
☐	☒	The statistical test(s) used AND whether they are one- or two-sided<br>*Only common tests should be described solely by name; describe more complex techniques in the Methods section.*
☒	☐	A description of all covariates tested
☐	☒	A description of any assumptions or corrections, such as tests of normality and adjustment for multiple comparisons
☐	☒	A full description of the statistical parameters including central tendency (e.g. means) or other basic estimates (e.g. regression coefficient) AND variation (e.g. standard deviation) or associated estimates of uncertainty (e.g. confidence intervals)
☒	☐	For null hypothesis testing, the test statistic (e.g. *F*, *t*, *r*) with confidence intervals, effect sizes, degrees of freedom and *P* value noted<br>*Give P values as exact values whenever suitable.*
☒	☐	For Bayesian analysis, information on the choice of priors and Markov chain Monte Carlo settings
☒	☐	For hierarchical and complex designs, identification of the appropriate level for tests and full reporting of outcomes
☒	☐	Estimates of effect sizes (e.g. Cohen's *d*, Pearson's *r*), indicating how they were calculated

Our web collection on statistics for biologists contains articles on many of the points above.

Software and code

Policy information about availability of computer code

Data collection	The 220 kHz ExAblate Neuro system type 2.0 software version 7.0 (InSightec, Israel) was used for all MR-guided focused ultrasound procedures (targeting, planing and sonications) and data collection.
Data analysis	The Statistical Parametric Mapping (SPM) software version 12 was used for the analysis of motor task fMRI.<br>FreeSurfer (https://surfer.nmr.mgh.harvard.edu/) was used for volume estimation of the precentral gyrus.

For manuscripts utilizing custom algorithms or software that are central to the research but not yet described in published literature, software must be made available to editors/reviewers. We strongly encourage code deposition in a community repository (e.g. GitHub). See the Nature Research guidelines for submitting code & software for further information.

Data

Policy information about availability of data

All manuscripts must include a data availability statement. This statement should provide the following information, where applicable:

- Accession codes, unique identifiers, or web links for publicly available datasets
- A list of figures that have associated raw data
- A description of any restrictions on data availability

The authors declare that all the data supporting the findings of this study are available within the paper and its supplementary information files. Study protocol, de-identified individual-participant data and data dictionary are available from the corresponding author upon request.

Field-specific reporting

Please select the one below that is the best fit for your research. If you are not sure, read the appropriate sections before making your selection.

☒ Life sciences ☐ Behavioural & social sciences ☐ Ecological, evolutionary & environmental sciences

For a reference copy of the document with all sections, see nature.com/documents/nr-reporting-summary-flat.pdf

Life sciences study design

All studies must disclose on these points even when the disclosure is negative.

Sample size	A cohort of four patients was planned for this early-phase trial. This sample size is typical for first-in-human surgical trials, where the objective is to demonstrate preliminary safety and technical feasibility. Given the small sample size, classic frequentist hypothesis testing based on p values was avoided to prevent type 1 errors and misleading inferences in this study which is underpowered to ascertain a mild or moderate treatment effect.
Data exclusions	No data were excluded from the analyses.
Replication	Standardized sonication parameters and power ramp tests were performed in all subjects. These parameters were validated previously by Lipsman et al. 2018 (Nat Commun. 2018; 9: 2336) and Mainprize et al. 2018 (Sci Rep. 2019 Jan 23;9(1):321). Reproducible blood-brain barrier opening was demonstrated as new gadolinium enhancement in all targeted areas.
Randomization	This is a single-armed study.
Blinding	This is an open-label study.

Reporting for specific materials, systems and methods

We require information from authors about some types of materials, experimental systems and methods used in many studies. Here, indicate whether each material, system or method listed is relevant to your study. If you are not sure if a list item applies to your research, read the appropriate section before selecting a response.

Materials & experimental systems

n/a	Involved in the study	
☒	☐	Antibodies
☒	☐	Eukaryotic cell lines
☒	☐	Palaeontology
☒	☐	Animals and other organisms
☐	☒	Human research participants
☐	☒	Clinical data

Methods

n/a	Involved in the study	
☒	☐	ChIP-seq
☒	☐	Flow cytometry
☐	☒	MRI-based neuroimaging

Human research participants

Policy information about studies involving human research participants

Population characteristics	Right-hand dominant, male or female volunteers aged ≥18 years were enrolled in the study. Each was diagnosed with laboratory-supported probable, clinically probable or definite ALS according to the revised El Escorial criteria. For safety reasons, participants had slow vital capacity (SVC) ≥50% for predicted age and body habitus and severe left hand or leg weakness.
Recruitment	Volunteers who contacted the study coordinator were pre-screened regarding their eligibility. All four volunteers who provided written informed consent and underwent the screening procedures were enrolled in the study. Efforts were made to balance sexes in this study.
Ethics oversight	The study was approved by the Research Ethics Board at Sunnybrook Health Sciences Centre (study number 453-2016) and Health Canada (ITA# 270370and CTA#207434).

Note that full information on the approval of the study protocol must also be provided in the manuscript.

Clinical data

Policy information about clinical studies
All manuscripts should comply with the ICMJE guidelines for publication of clinical research and a completed CONSORT checklist must be included with all submissions.

Clinical trial registration	Clinicaltrials.gov NCT03321487
Study protocol	The study protocol is available from the corresponding author on request.
Data collection	Data collection was obtained at the Sunnybrook Focused Ultrasound Centre of Excellence and ALS Clinic, University of Toronto. The study was initiated in April 2018 and the data collection was completed in October 2018.
Outcomes	The primary outcome was safety and feasibility of transient BBB opening by MRgFUS in the primary motor cortex. Safety was measured as the occurrence and severity of device- and procedure-related, clinical or radiologic adverse events. Feasibility was qualitatively defined as detectable gadolinium enhancement signal into the sonicated targets on T1-weighted imaging immediately post-procedure, and resolution of the enhancement by the next day (reversibility criterion).<br>Secondary outcome measures: feasibility was quantified by the gadolinium signal intensity ratio within a region of interest (ROI) in the targeted area, normalized to the contralateral, unsonicated mirrored ROI. Exploratory measures (modified Ashworth Scale for spasticity, Montreal Cognitive Assessment [MoCA], ALS Functional Rating Scale Revised [ALSFRS-R], and electroencephalography [EEG]) were collected at baseline and at day 30. The final study event was a remote visit 60 days after the procedure to collect adverse events and ALSFRS-R.

Magnetic resonance imaging

Experimental design

Design type	Block design task functional MRI, volumetric estimates on T1w MRI
Design specifications	One trial per subject (4 minutes 28 seconds). Each trial consists of 6 blocks of repetitive movement to written command projected on a screen, interspersed with rest blocks (i.e. crosshair fixation), each block lasts 10 seconds.
Behavioral performance measures	each subject was instructed immediately prior to the MRI on performing the motor task (either hand squeeze or foot tap) and had opportunity to practice, performance was assessed informally by verbal feedback from subjects.

Acquisition

Imaging type(s)	Functional MRI, T1-w MRI
Field strength	3T
Sequence & imaging parameters	functional: 3D fast spoiled gradient echo, TE = 30ms, TR = 2000ms, flip angle = 70, matrix size = 64 x 64, 4mm thickness<br>structural: 3D fast spoiled gradient echo, TE = 2.94 ms, TR = 7.65 ms, and matrix size = 265x265, 1mm thickness
Area of acquisition	Whole brain

Diffusion MRI ☐ Used ☒ Not used

Preprocessing

Preprocessing software	Functional analysis: Statistical Parametric Model (SPM) version 12, spatial realignment to first functional volume, reslicing using 4th degree B-spline interpolation, slice-time correction, co-registration functional to spatial scans using normalized mutual information, normalization to ICBM152, spatial smoothing of functional volumes with 6mm Gaussian kernel<br>Volumetric analysis FreeSurfer, longitudinal, surface-based pipeline, documented and available online at http://surfer.nmr.mgh.harvard.edu/. Details of this specific analysis was described by Reuter et al. Neuroimage 2012.
Normalization	Default procedures in SPM12 for normalization with 4th degree B-spline interpolation
Normalization template	Default ICBM152 template was used
Noise and artifact removal	Movement-related artifacts were modeled as regressors by realignment parameters (X, Y, Z displacement, and X, Y, Z rotation) generated by SPM alignment procedure
Volume censoring	No specific scrubbing procedure used

Statistical modeling & inference

Model type and settings	First level analysis only
Effect(s) tested	Simple [1 -1] contrast of movement blocks to rest blocks

Specify type of analysis: ☒ Whole brain ☐ ROI-based ☐ Both

Statistic type for inference (See Eklund et al. 2016)	Individual activation maps were generated from voxel-wise t-statistics after FWE corrected $p < 0.05$
Correction	FWE corrected $p < 0.05$

Models & analysis

n/a	Involved in the study
☒	☐ Functional and/or effective connectivity
☒	☐ Graph analysis
☒	☐ Multivariate modeling or predictive analysis
